# Multi-tissue neocortical transcriptome-wide association study implicates 8 genes across 6 genomic loci in Alzheimer’s disease

**DOI:** 10.1186/s13073-021-00890-2

**Published:** 2021-05-04

**Authors:** Jake Gockley, Kelsey S. Montgomery, William L. Poehlman, Jesse C. Wiley, Yue Liu, Ekaterina Gerasimov, Anna K. Greenwood, Solveig K. Sieberts, Aliza P. Wingo, Thomas S. Wingo, Lara M. Mangravite, Benjamin A. Logsdon

**Affiliations:** 1 Sage Bionetworks, Seattle, WA USA; 2 Department of Neurology, Emory University School of Medicine, Atlanta, GA 30322 USA; 3 Division of Mental Health, Atlanta VA Medical Center, Decatur, GA USA; 4 Department of Psychiatry, Emory University School of Medicine, Atlanta, GA USA; 5 Department of Human Genetics, Emory University School of Medicine, Atlanta, GA 30322 USA; 6 Cajal Neuroscience, 1616 Eastlake Avenue East, Suite 208, Seattle, WA 98102 USA

**Keywords:** Alzheimer’s disease, TWAS, FUSION, GWAS, Dementia, Neurodegeneration, AMP-AD

## Abstract

**Background:**

Alzheimer’s disease (AD) is an incurable neurodegenerative disease currently affecting 1.75% of the US population, with projected growth to 3.46% by 2050. Identifying common genetic variants driving differences in transcript expression that confer AD risk is necessary to elucidate AD mechanism and develop therapeutic interventions. We modify the FUSION transcriptome-wide association study (TWAS) pipeline to ingest gene expression values from multiple neocortical regions.

**Methods:**

A combined dataset of 2003 genotypes clustered to 1000 Genomes individuals from Utah with Northern and Western European ancestry (CEU) was used to construct a training set of 790 genotypes paired to 888 RNASeq profiles from temporal cortex (TCX = 248), prefrontal cortex (FP = 50), inferior frontal gyrus (IFG = 41), superior temporal gyrus (STG = 34), parahippocampal cortex (PHG = 34), and dorsolateral prefrontal cortex (DLPFC = 461). Following within-tissue normalization and covariate adjustment, predictive weights to impute expression components based on a gene’s surrounding *cis*-variants were trained. The FUSION pipeline was modified to support input of pre-scaled expression values and support cross validation with a repeated measure design arising from the presence of multiple transcriptome samples from the same individual across different tissues.

**Results:**

*Cis*-variant architecture alone was informative to train weights and impute expression for 6780 (49.67%) autosomal genes, the majority of which significantly correlated with gene expression; FDR < 5%: *N* = 6775 (99.92%), Bonferroni: *N* = 6716 (99.06%). Validation of weights in 515 matched genotype to RNASeq profiles from the CommonMind Consortium (CMC) was (72.14%) in DLPFC profiles. Association of imputed expression components from all 2003 genotype profiles yielded 8 genes significantly associated with AD (FDR < 0.05): APOC1, EED, CD2AP, CEACAM19, CLPTM1, MTCH2, TREM2, and KNOP1.

**Conclusions:**

We provide evidence of cis-genetic variation conferring AD risk through 8 genes across six distinct genomic loci. Moreover, we provide expression weights for 6780 genes as a valuable resource to the community, which can be abstracted across the neocortex and a wide range of neuronal phenotypes.

**Supplementary Information:**

The online version contains supplementary material available at 10.1186/s13073-021-00890-2.

## Background

Alzheimer’s disease (AD) is a progressive, incurable neurodegenerative disease accounting for 60–70% of all dementia diagnoses [1], currently affecting 5.8 million Americans and projected to grow to 13.8 million diagnoses by 2050 [2]. Late-onset Alzheimer’s disease (LOAD) comprises over 95% of AD diagnoses and is composed of a diverse, largely unknown set of etiologies [3]. The mechanisms of AD remain insufficiently explained, despite clear Alpha-Beta plaque and Tau neurofibrillary tangle neuropathology. Additionally, known highly penetrant genetic variants from familial-based cohorts with early-onset Alzheimer’s disease (EOAD) implicate genes such as APP, PSEN1, and PSEN2. Despite clear pathology and known risk factors, AD therapeutic clinical trials have consistently failed [4, 5]. Elucidating the mechanisms by which AD genetic risk loci contribute to AD disease and disease progression is instrumental in the development of future impactful therapeutics.

While genome-wide association studies (GWAS) identified some candidate loci associated with AD risk, genes targeted through *cis-*genetic risk factors remain unclear [6, 7]. Likewise, postmortem bulk-cell transcriptomics show vast expression changes across multiple neocortical regions; however, it remains difficult to identify which are driving AD from expression changes merely caused by the disease state of widespread cell death and tissue degeneration [8, 9]. Transcription-wide association studies (TWAS) help provide this associative bridge and mechanistic direction of effect between genotype, transcript, and disease status [10]. TWAS leverages the cis-genotype region surrounding an expressed gene to predict the *cis*-heritable component of a gene’s expression, which in turn can be associated to disease status using GWAS summary statistics.

We modified the FUSION pipeline [10], which deploys blup, bslmm, lasso, top1, and enet models to predict gene expression from *cis*-variants within 1 MB of a given gene to train weights and impute expression for 6780 (49.67%) autosomal genes from matched genotypes and RNA-Seq profiles from dorsolateral prefrontal cortex (DLPFC), temporal cortex (TCX), prefrontal cortex (PFC), superior temporal cortex (STG), inferior temporal gyrus (IFG), and parahippocampal gyrus (PHG) provided by the Accelerating Medicines Partnership - Alzheimer’s Disease (AMP-AD) consortia. Imputed gene expression was validated in CommonMind Consortium (CMC) DLPFC. Using our trained models, we imputed gene expression into a large, recent GWAS cohort [6] to identify genes showing differential predicted gene expression between AD patients and controls. Following correction multiple testing, joint conditional probability testing (JCP), and summary Mendelian randomization (SMR), we discover eight candidate AD risk genes APOC, EED, CD2AP, CEACAM19, CLPTM1, MTCH2, TREM2, and KNOP1.

Expanding associated genes into gene sets using co-expression yielded enrichments for specific cell-type marker sets particularly microglial, oligodendrocyte, and astrocyte cell populations and cellular functions such as protease binding, myeloid, and leukocyte regulation/activation, regulation lipid/lipoprotein, RNA splicing, and steroid regulation. We identify 8 genes across six distinct genomic loci associated to AD through gene expression attributable to their *cis*-genetic variation. Trained gene expression weights are a community resource which can be abstracted to multiple phenotypes and gain further insights from large genotyped cohorts to maximize the informativeness of invaluable and rare patient material [11]. To this end, we provide a valuable resource to the community in the form of predictive gene expression weights which can be leveraged across a wide range of neurological phenotypes.

## Methods

### Ancestry analysis

Ancestry analysis and clustering was performed to identify individuals with northern and western European ancestry from the combined ROSMAP, Mayo, and MSBB cohort. Briefly, phase I 1000 Genomes data [12] was filtered for YRI, CHB, JPT, and CEU ancestral populations. Genotype data was combined across MSBB, Mayo, and ROSMAP cohorts, filtered for 1000G overlapping SNPs, and combined with 1000G data from the four reference ancestral populations. PCA was performed using Plink (v1.9) on SNPs passing filtering: minor allele frequency > 1%, missingness < 0.1, maximum minor allele frequency < 40%, and independent pairwise linkage filter window of 50 Kb at 5 Kb steps and an r-squared threshold of 0.2. PCA results were visualized along PC1 (50.7%) and PC2 (31.6%). Genotype clustering to identify clustered genotype profiles was performed with the R package GemTools [13, 14]. The clustering of genotype profiles was performed on all PCs describing greater than 1% of variation. Genotypes were recoded for GemTools clustering in plink similar to the PCA but with the added flags: --recode12, --compound-genotypes, --geno 0.0000001. Ancestrally matched CEU samples were identified as any sample genotypes belonging to one of the three out of eight clusters to which contained 1000G reference CEU individuals (Additional file 1: Fig. S1).

### Defining the TWAS training set

**Fig. 1:**
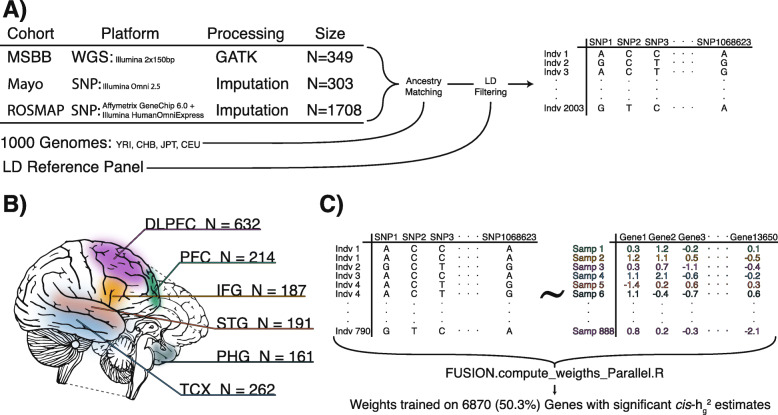
Experimental design. **a** Sample cohorts from MSBB, Mayo, and ROSMAP were combined across platforms by consensus SNP variant sites. Ancestry analysis was performed, and sites within the 2003 CEU ancestrally matched populations were filtered for consensus with the LD reference panel. **b** RNA-Seq samples originated from 6 distinct neocortical regions. **c** The training set data for training TWAS weights consisted of 888 RNA-Seq samples matched to 789 individual variant profiles

Genotype data for 2360 individuals was combined across MSBB (*N* = 349), Mayo (*N* = 303), and ROSMAP (*N* = 2360) cohorts (*Specific details see: supplementary methods sections 1–2*). CEU 1000G reference individuals were present within the top 3 clusters (Additional file 1: Fig. S1c) which contained 2003 individuals across all cohorts (Additional file 1: Fig. S1[b,d]). Broader admixture was observed in the MSBB cohort compared to ROSMAP and Mayo representative of study recruitment and population (Additional file 1: Fig. S1[e-h]). After filtering for variants present within the LD-Reference panel, the final European ancestry filtered population of 2003 individuals was represented by 1,069,623 variants (Fig. 1a).

Expression profiling from high-throughput sequencing from all three studies: ROSMAP (DLPFC *N* = 632), Mayo (TCX *N* = 262), and MSBB (PHG *N* = 161, IFG *N* = 187, STG *N* = 191, FP *N* = 214) were normalized within study. For description of the individual expression data and processing, see supplementary methods (Additional file 1: Supplementary methods sections 3–4). Iterative normalization was performed to regress significant covariates for individual studies including, but not limited to, diagnosis, age at death, sex, and post mortem interval (Additional file 2: Table S1). Diagnosis was regressed as is common practice in TWAS and eQTL studies as genetic allele risk has been found to be largely condition-independent [15, 16]. Post quality control and normalization, 888 RNA-Seq samples from all three studies (ROSMAP (DLPFC *N* = 481), Mayo (TCX *N* = 248), and MSBB (PHG *N* = 34, IFG *N* = 41, STG *N* = 34, FP *N* = 50) were matched to 790 CEU ancestrally matched individuals (Fig. 1b, c, Table S4-S5). Genotypes were represented multiple times for a subset of MSBB individuals to create the 888 unique genotype-expression pairs. Among the 60 ancestrally matched MSBB genotypes, 15 were profiled in all four tissues, 22 were profiled in three tissues, 10 were profiles in 2 tissues, and 13 were only profiled in one of the four tissues. The final training set contained 275 designated AD cases, 181 designated controls, and 432 genotype-phenotype profiles which failed to meet a diagnostic case/control status from neuropath and cognitive assessments.

### Training TWAS weights

Multiple regions per-individual were assayed, in such cases both expression profiles were paired to the individual’s genotype in the training set. The resulting training set consisted of 790 ancestrally clustered genotypes matched to 888 normalized, scaled RNA-Seq profiles with diagnosis regressed. Weights predicting gene expression were trained on matched genotype-RNA-Seq profiles and then used to impute the expression components of all 2003 genotypes in our CEU ancestrally defined cohort. The FUSION software [10] was modified to accommodate the presence of multiple RNA-Seq profiles across different regions for the same individual by ensuring that all samples from a given individual were present within a single cross-validation fold during training and model optimization. The FUSION pipeline script was altered cross-validating cohorts of multiple-samples per-individual, this capacity also ensured that each cross-fold validation sample was within 5% of the size every other fold and could accept pre-scaled expression values with specification of an additional flag *--scale 1*. Gemma (v0.98.1) calculated the *cis*-heritability of scaled expression using all SNPs denoted in the LD-Reference panel and within 1 MB window centered on the gene’s TSS for all 13650 autosomal genes. Weights were trained using all five TWAS models (blup, bslmm, lasso, top1, enet) for the 6780 genes with a significant *cis*-heritability (*p* < 0.01). To support the computational requirements of all five models, the FUSION software was altered to run on 5 threads and run in 14x parallel across an AWS c5.18xlarge (72 core 144 MB) EC-2 instance. All supporting files for training weights are available on Synapse [17] as well as trained weights in RData files which can be used to impute expression components with a user provided genotype profile.

### Expression imputation and TWAS gene associations

**Fig. 2:**
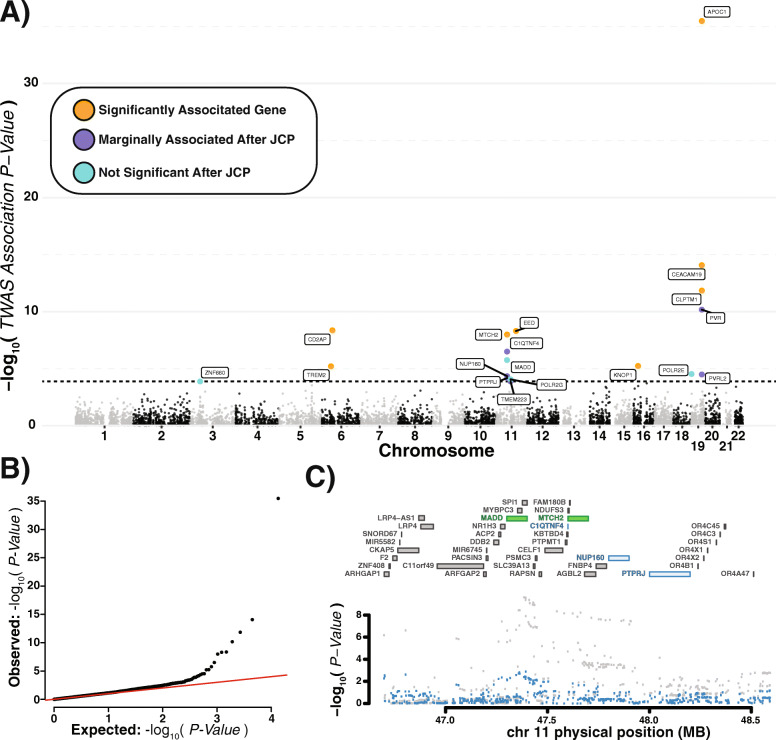
Transcriptome-wide association study results. **a** Log 10 TWAS association *p* values by gene shown by genomic location are indicated in black and grey. Features passing initial correction for multiple comparisons (above the dotted line), but marginally significant after joint conditional probability (JCP) are shown in purple. Those features which are no longer significant after JCP are shown in light blue, while genes surviving JCP are shown in yellow. **b** QQplot of all TWAS *p* values. **c** An example plot of a region tested for JCP. The candidate genes found to be marginally significant, NUP160 and PTPRJ, are colored blue while those found to be jointly significant, MADD and MTCH2, are colored green (upper), while individual SNP *p* values are colored grey before and blue after conditioning (lower)

The heritable component of gene expression was imputed for 6780 genes with trained weights. Despite being trained on only the 790 individuals with 888 matched RNA-Seq profiles, expression components based on cis-genotype were able to be imputed for the entire combined Mayo, MSBB, and ROSMAP genotype cohort of 2003 CEU ancestry-matched individuals on an AWS r3.8xlarge (32 core 144 MB) EC-2 instance. While expression components were able to be imputed for 2003 genotypes, weight training was only able to be performed on the matched 888 genotype to expression profiles. Association of AD cases versus control using Kunkle et.al GWAS summary statistics [6] was performed with the FUSION.assoc_test.R script [10]. AD case and control designation was specified with strict neuropathological diagnosis criteria cutoffs as specified in supplementary table 5. Only 635 out of 2003 ancestrally matched individuals from the combined Mayo, MSBB, and ROSMAP genotypes were designated as AD cases (*N* = 404) or controls (*N* = 231) (Fig 2a).

### CMC DLPFC validation

CMC count data was ingested and processed similar to the AMP-AD transcriptome data. An iterative normalization model was deployed to identify significant covariates and regress them from the expression data before scaling the data (Additional file 2: Table S2). Genotype data was profiled with Affymetrix GeneChip Mapping 5.0 K Array and a custom version of the Illumina Infinium CoreExome-24 v1.1 BeadChip (#WG-331-1111). Raw data was filtered to remove SNPs with: zero alternate alleles, MAF < 1%, genotyping rate < 0.95, Hardy-Weinberg *p* value < 1 × 10^−6^, and individuals with genotyping rate < 0.95. Imputation was performed using eagle, Minimac, and the HRC Reference Panel [18]. Imputed variant data was filtered for SNPs present in the LD reference panel using Plink (v1.9). CMC data was withheld from training gene weight models for the purpose of validating gene weights in an independent cohort, blinded from the training models. Expression values were imputed, and Kendall correlation values were calculated comparing imputed gene expression to the scaled, assayed expression values. Correlation test values were FDR corrected for the number of matched comparisons *N* = 6643.

### JCP, SMR, and COLOC analysis

To assess the independence of these associations within their respective 1 MB windows, JCP testing was performed [17, 19]. In order to replicate our AD associations, SMR [17, 20] was run on all 6780 genes with weights and analyzed for TWAS preliminary hits. Correction for 18 multiple comparisons was applied for replication of associated genes (Additional file 2: Table S2). JCP analysis was run on all candidate hit regions with FUSION.post_process.R to disentangle signal within regions with multiple significant genes by examining the probability that multiple associations occur simultaneously (jointly). This analysis module helps identify genes which are associated irrespective of surrounding genes (Marginal) from those which rely on surrounding loci (Conditional) [10, 19, 21–23]. COLOC analysis was carried out to examine the probabilities that the expression-AD phenotype signals for the remaining targets were driven by the same underlying genomic variant etiology [24–26] (Additional file 2: Table S6).

### GWAS enrichments

In order to examine whether the subset of genes with trained TWAS weights were enriched with variants more likely to regulate gene expression within their centered 1 MB window compared to brain expressed genes without trained weights or the rest of the genome, summary statistics for both Kunkle et al. [6] and Styrkarsdottir et al. [6, 27] were partitioned into 3 groups: SNPs that were within 1 MB of the TSS of genes which had trained TWAS weights, SNPs that were within 1 MB of the TSS of Autosomal genes which did not have trained TWAS weights, and autosomal SNPs that were not within 1 MB of an expressed gene (Intergenic). Wilcoxon rank-sum tests were performed comparing *p* value distributions between SNPs near genes with trained weights versus those without, as well as within and intergenic regions (Additional file 2: Fig. S6).

### Gene set expansion and cell type analysis

**Fig. 3:**
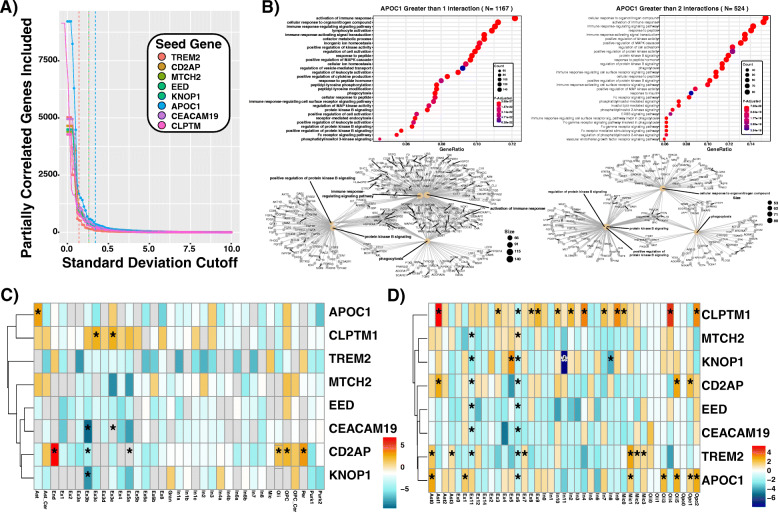
Cell-specific and cell process enrichment analysis. **a** Gene-set cutoffs of partial correlation to associated gene as a function of standard deviations away from the mean partial correlation. These are the cutoffs used for cell type specific enrichments seen in **c** and **d**. **b** EnrichR cell process enrichments of APOC1 expanded gene set of the 50 highest partially correlated genes and then expanded to all protein interaction partners from the pathway commons database which are represented more than once (Left) and twice (Right). **c** Cell type specific enrichments of expanded gene sets using Lake et. al cell type specific marker gene sets. Grey denotes zero overlapping genes between gene set and cell type-marker gene set significance in a grey square infers depletion from the expected overlap by chance. **d** Cell type specific enrichments of expanded gene sets using Mathys et. al cell type specific marker gene sets

To examine functional enrichments and identify potential processes driving AD, we used coexpression to build out a gene set focused on each TWAS associated gene. We first wanted to consider the possibility that another gene within the 1 MB window was co-regulated with our identified gene of interest and therefore could be the causative gene. For all 6 tissues, Variable Bayes Spike Regression [28, 29] was used to calculate the bootstrapped partial regression of each TWAS associated gene to all other genes. If the mean correlation of a gene within the 1 MB *cis*-regulatory region was greater than 0.1, the additional gene was also correlated to all other transcribed genes for all six tissues. The only gene that met this criterion and was added for further analysis was APOE, as it resides within the 1 MB window surrounding APOC1. For each gene or in the case of the APOC1 locus, APOC1 and APOE, the mean partial correlations across all 6 neocortical tissues were used to enrich for co-expressed functionally related genes. The elbow plots indicating the decay of included genes as the standard deviation of mean partial correlations moves away from the centered mean of zero. This was used to draw cutoffs for inclusion into the expanded gene set at values of 0.7 (TREM2), 1.3 (KNOP1, CD2AP, MTCH2, EED), and 1.7 (APOC1) (Fig. 3a).

For cell-type enrichments, TWAS expanded co-expression gene sets were analyzed for enrichment in cluster-specific marker genes from Lake et al. [30] and Mathys et al. [31]. Odds ratios were calculated with the percent overlap of a gene set and cell-type specific marker gene list divided by the expected percentage of overlap. Significance was calculated with a two-sided exact binomial test, FDR < 0.05 corrected for 35 (Lake) and 41 (Mathys) comparisons.

For cell process enrichments, a multiscale gene set expansion was employed, foundationally based on small scale enrichment of expanding the candidate TWAS gene to the top 50 bootstrapped partial regression inclusion statistics averaged across all tissues. The second expansion was performed by leveraging pathway commons protein-protein interaction databases from Pathway Commons [32]. All pairwise gene-gene interactions containing a gene member with the initial gene set expansion were extracted, and the total gene set was expanded into a broad and narrow range gene set by including any gene which appears more than once (broad) or more than twice (narrow). Ensembl gene IDs were translated to gene symbols and filtered for brain relevance by requiring them to be expressed in at least one brain region. These gene sets were then submitted to EnrichR [33, 34] to find cell process enrichments with the background set being any gene set to the list of all genes expressed in any one of the 6 brain regions analyzed.

## Results

### Training and validating TWAS weights

A total of 6818 (49.95%) genes had significant *cis*-heritability estimates (*p* value < 0.01) and therefore had weights trained for them; only 6780 of 13650 (49.67%) could have non-zero variance expression components imputed into the CEU ancestrally matched genotype cohort of 2003 individuals (Additional file 1: Fig. S.2). Imputed expression components based on *cis*-genotype contribution were correlated to actual expression values for all 888 matched genotype to RNA-Seq profiles which was the inclusion criteria of the training set. Kendall correlations were calculated for all 6780 imputed to actual gene expression, resulted in 6775 (99.93%; FDR < 0.05, *N* = 13650) imputed gene profiles significantly correlated with the actual expression and 6716 (99.06%) were significantly correlated after Bonferroni correction (Additional file 1: Fig. S.3a). The distribution of correlations was right-skewed towards one (*X* = 0.24 ± 0.09) (Additional file 1: Fig. S.3b). Comparing imputed expression components to actual gene expression for four representative weights (Additional file 1: Fig. S.5) confirmed that weights were not biased by tissue type or cohort, and our regression normalization and expression scaling coupled with changes to the FUSION trained weights specific to the continuous heritable expression difference across our training set (Additional file 1: Fig. S.4[a-d]).

CMC DLPFC data was used as an independent validation cohort to examine the extractability of the TWAS weights to other datasets. CMC data was comprised of 515 individually matched genotype to expression profiles with a set of 6756 expressed genes overlapping the trained weights, representing 99.6% of all trained weights. Kendall rank correlation values between imputed expression components and observed expression were right-skewed towards one (*X* = 0.13 ± 0.11) (Additional file 1: Fig. S.5b). FDR correction for multiple comparisons yielded 4874 (72.14%) genes with significant, positive correlations between imputed and actual DLPFC expression (Additional file 1: Fig. S.5a). Genes with a significant *p* value (FDR < 0.05) and positive correlation values all had correlation values greater than 0.061 (Additional file 1: Fig. S.5c).

Given the threshold of expression heritability required to train weights for a given gene, it could be expected that variants regulating gene expression would be enriched within 1 MB of genes with trained weights versus genes which did not meet the heritability threshold. We looked for enrichment of low *p* value SNPs within the 1 MB widow centered on genes with trained weights to test this assumption. Variants from Kunkle et al. [6] were binned into three groups (Additional file 1: Fig. S.6a). The first were the autosomal variants within the 1 MB window of genes which had trained weights; the second consisted of autosomal variants within the 1 MB window of genes which did not meet the 1% heritability threshold to have weights trained for them. The final group of variants was termed intergenic and consisted of all variants outside of a 1 MB window centered on any of the 13650 expressed genes in the training dataset regardless of whether predictive weights were able to be trained for the gene. Variants within 1 Mb of genes meeting the heritability threshold versus were significantly enriched in lower GWAS *p* values than those within 1 MB of genes below the heritability threshold (*p* < 2.22^−16^ Wilcoxon rank-sum) as well as intergenic variants (*p* < 3.70^−15^ Wilcoxon rank-sum). To confirm this result, the same analysis was performed with variants from Styrkarsdottir et al.’s [27] bone density GWAS analysis (Additional file 1: Fig. S.6b). This outgroup confirmed enrichment for associated variants within genes of higher heritability versus those of lower heritability (*p* < 2.22^−16^ Wilcoxon rank-sum) as well as intergenic variants (*p* < 2.22^−16^ Wilcoxon rank-sum). Despite potential edge cases, such as genes regulated from long distance LD, genes in MHC regions, and *trans*-regulatory effects, this analysis suggests that our weights are enriched for genes under a higher degree of *cis*-genetic modular control, although the rate at which linkage-disequilibrium affects SNP independence is unknown. The outgroup bone density GWAS dataset confirms that this enrichment is for genetic variants controlling gene expression irrespective of AD phenotype, and tissue context, supporting these weights as a general resource across neocortical regions for generalizable use across multiple phenotypes.

### Alzheimer’s disease TWAS

Imputed gene expression components were associated with AD through implementation of the FUSION pipeline. This analysis yielded 18 preliminarily significant associations across 8 regions after correction for multiple comparisons (FDR < 0.05) (Fig. 2a, Table 1). JCP testing resulted in dropping 6 preliminary associations as a result of marginal association (Fig. 2c, Fig. S7). FDR correction of the JCP *p* value resulted in dropping an additional 4 targets due to non-significance. Association of all remaining genes was further supported by SMR (Additional file 2: Table S3). The remaining 8 genes comprising 6 distinct non-overlapping 1 MB genomic regions and are significantly associated to AD after JCP and SMR with FDR corrected *p* values are as follows: APOC1 (JCP = 2.22e−22, SMR = 3.41e−4), EED (JCP = 3.373e−5, SMR = 2.50e−4), CD2AP (JCP = 2.96e−5, SMR = 2.66e−4), CEACAM19 (JCP = 3.27e−5, SMR = 1.00e−2), CLPTM1 (JCP = 4.04e−3, SMR = 2.58e−3), MTCH2 (JCP = 0.011, SMR = 3.32e−6), TREM2 (JCP = 0.021, SMR = 2.64e−3), and KNOP1(JCP = 0.039, SMR = 2.50e−4). All gene associations except MTCH2 and KNOP1 replicated and survived JCP testing when summary statistics from Jansen et al. were used instead of Kunkle et al*.* summary statistics.

**Table 1:**
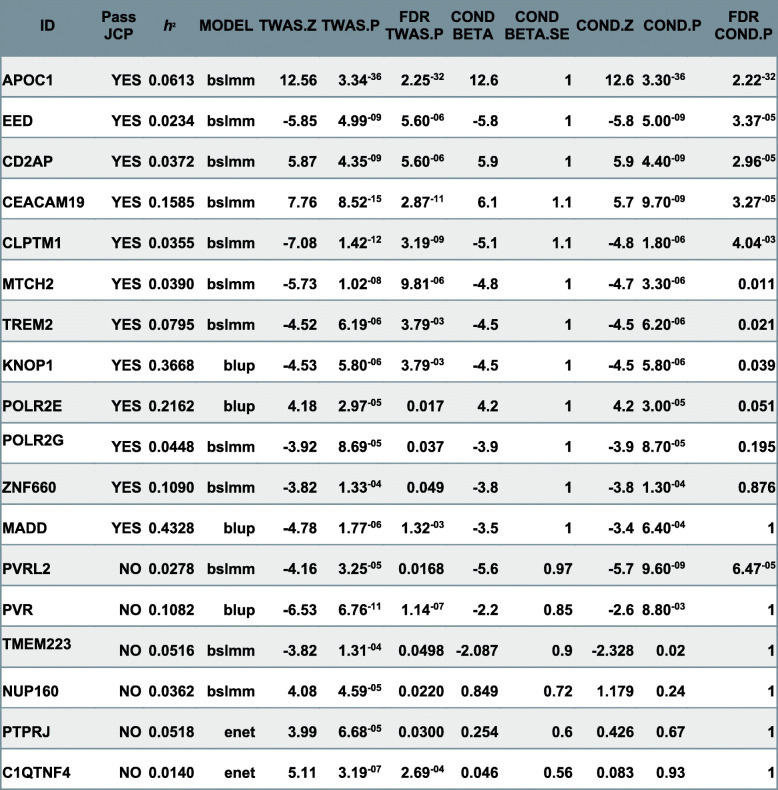
Heritability (*h*^2^), best performing model, before and after JCP *Z* values and *p* values for all initially significant AD-associated genes. Blue denotes those only marginally significant after JCP, while green represents independently significant genes

### Cell type specificity and pathway enrichment

Expanded gene sets of genes co-expressed to TWAS nominated AD-associated genes were built empirically for each region’s gene set based on membership decay given increasing standard deviation cutoff (Fig. 3a, see the “Gene set expansion and cell type analysis” section). Distinct cell type enrichments were seen comparing gene sets to the cell type-specific marker lists observed in Lake et al. [30] (Fig. 3c) and Mathys et al. [31] (Fig. 3d). APOC1 and TREM2 coexpression sets were enriched in astrocyte and microglial markers respectively. The CD2AP gene set was enriched within endothelial, pericytes, and oligodendrocyte expression signatures. KNOP1, CEACAM19, MTCH2, and EED co-expression gene set was not enriched within any of the cell-type specific expression sets, but showed sporadic enrichments in neuronal cell types (Fig. 3c, d). CLPTM1 was enriched across neuronal populations, oligodendrocytes, and astrocytes (Fig. 3c, d). Notably, the largest two single-cell marker gene sets, derived from excitatory neuronal populations, Ex6 and Ex11, were more prone to enrichment. This could have been due to their containing significantly more genes than the other gene sets, which contain 850 (Ex6) and 747 (Ex11).

Cell process-enriched gene sets were built with a multiscale approach combining pairwise inclusion statistics with protein-protein interactions. Both a wide inclusion cutoff and a more stringent inclusion cutoff produced a permissive and conservative gene set for each candidate gene (see the “Gene set expansion and cell type analysis” section). APOC1 was enriched for multiple immune response signaling pathways, phagocytosis, and immune activation consistently across both enriched gene sets (Fig. 3b). CD2AP was enriched for cellular responses to lipids, protein localization, and responses to multiple molecular compounds (Additional file 1: Fig. S.8). EED, CLPTM1, and CEACAM19 were consistently enriched for RNA, mRNA processing, RNA splicing, and RNA translation processes. In addition to high amounts of transcription relevant overlap between the three candidate genes, distinction of enrichments of viral gene expression response (EED), protein catabolic processes for (CLPTM1), and myeloid/megakaryocyte differentiation (CEACAM19) were observed (Additional file 1: Fig. S.9,S13-14). MTCH2 was enriched for purine, deoxynucleotide, and ribo-deoxynucleotide metabolism (Additional file 1: Fig. S.10). KNOP1 was the smallest gene set within both the permissive and conservative cutoff groups. Nominal enrichments for mitotic cell phase transition and Wnt signaling point to a potential a role in cell-cycle progression (Additional file 1: Fig. S.11). TREM2-expanded gene sets showed enrichment for immune response activation, T cell and leukocyte activation, and cell motility and phagocytosis (Additional file 1: Fig. S.12).

## Discussion

We trained predictive models to impute gene-expression components attributable to *cis*-variation across multiple neocortical tissues using a well-powered training set compared to the field of TWAS studies [35, 36]. This is the first pan-cortical analysis and is broadly abstractable throughout the neocortex, providing a valuable resource to investigate a multitude neurological conditions and disorders. While it is commonly accepted practice in TWAS studies to combine tissues to enhance the power of predictive weight models, specifically including a range of neocortical structures relevant to AD, we sought to specifically identify drivers of AD capable of working across these diverse regions while also maximizing the sample size of valuable neuronal tissues derived RNA-Seq samples. Beyond AD, there are a number of neuropsychiatric conditions schizophrenia, depression, and ASD to name a few, which affect the neocortex as a whole. As AD disease status was regressed, a common practice in TWAS and eQTL studies from training set expression data, our trained weights represent a valuable resource capable of giving insight into the mechanisms of neocortical phenotypes [15, 16].

We leveraged these weights to perform a TWAS between Alzheimer’s cases and controls, revealing 8 candidate genes across 6 distinct regions which passed multiple filters for significance after correction for JCP and SMR replication. We used the Jansen et al. AD GWAS study [7] to replicate our findings, confirming six of the eight genes with this data set. The two genes that failed to replicate after correction for multiple comparisons were MTCH2 and KNOP1, which were not identified in Jansen et al. [7], indicating that our methodology is consistent with the input GWAS statistics. Importantly, as imputed expression is dependent on genotype, gene expression is associated with AD directly through underlying regulatory *cis*-genetic risk factors. These methods can have difficulty in training expression weights for relevant genes which have a high variance or are regulated under trans-regulation such as miRNA mediated transcript decay. Likewise, even though we detect a strong signal for TREM2, a known risk factor predominantly expressed in microglia, a low representation cell type, there remains a possibility that our signal is biased towards cell types of greater representation. While bulk RNA sequencing is potentially confounded by cell proportion, our signal supports previous work implicating microglial contribution to AD pathology and disease mechanism. As new single-cell genomics technology increases the available datasets on human postmortem cell lineages in future work will be needed to focus on genotype to expression linkage methods such as TWAS in this context. Larger sample sizes and a wider array of neocortical tissue types may help mitigate these difficulties; however, the vital nature of these biospecimens makes it understandably difficult to address completely. Co-regulation within the *cis*-genetic window is a possible confounder in any TWAS analysis, as a more stably expressed co-regulated gene could possibly produce a more robust association than the true causative disease-linked gene [37]. This effect, distributed across a region, can coordinately drive AD disease mechanism through multiple genes within the locus. With the exception of APOE being highly coexpressed with APOC1, no other gene within a window is appreciably coexpressed with the TWAS candidate gene, granting confidence in our 8 candidate genes. The inclusion of a diverse set of brain regions into the training set may disrupt co-regulation based on tissue-specific expression and differential disease impact across brain regions could introduce variance into the model. This is a particular strength of our study. However, as AD pathology affects all of our included regions, fundamental driving risk genes could be expected to be identified across our neocortical tissue set. As more genetic, molecular, and biological process factors are associated with AD, methods such as TWAS represent a vital way to connect lines between these tiers of evidence, building a clearer picture of AD mechanism throughout the brain. While it is important to consider the whole locus in the context of our TWAS associations, this evidence supports our associated genes.

### CD2AP, Chr6

JCP analysis supports CD2AP as the most likely linked gene within this locus, an observation that is consistent with broader biological investigations implicating upregulation of CD2AP in AD. Colocalization analysis resulted in a 99% probability that GWAS association signal and functional TWAS signal for CD2AD originate from shared causal variants (Additional file 2: Table S6). All four of the largest GWAS studies performed looking at AD genetic associations have found variants that point to CD2AP [6, 7, 38, 39]. The biological role of CD2AP involves dendritic targeting of APP to the intraluminal vesicles (ILV), which functions as the post-synaptic lysosomal complex, for degradation [40]. Targeting APP to the ILV leads to proteolytic clearance, decreases the shared time spent with BACE within the endosomal complex, and results in decreased amyloid secretion. CD2AP knockdown impairs targeted APP degradation, allowing APP and BACE to co-exist within the early endosomal compartment and increasing amyloid production [40]. Accordingly, CD2AP over-expression drives APP localization from Rab5+ early endosomes to Rab7+ late endosomes, leading to lysosomal degradation and decreased amyloid secretion [41]. Autosomal dominant AD mutations, associated with EOAD, resulted in enlargement of the early endosomal compartment and elevated levels of the BACE-cleaved APP carboxy-terminal fragment (CTFbeta) in cortical neurons derived from IPSCs [42]. This work supports the emerging viewpoint that perturbations in endocytosis play a fundamental role in AD biology.

Towards its role in AD, CD2AP operates in concert with BIN1 as a functional regulation mechanism. While CD2AP promotes trafficking towards ILV for degradation in the dendrites, BIN1 targets BACE from the early endosome back to the cell surface within axons, preventing the colocalization of APP and BACE in endosomal compartments and decreasing the levels of secreted amyloid. While it may be too narrow a perspective to look only at amyloid biogenesis for linkage with disease mechanism, it provides one plausible framework for consideration. Expanded gene set enrichment of CD2AP identifies a range of processes implicated in both the regulation of tissue development as well as responses to lipid and organic cyclic compounds (Additional file 1: Fig. S.8). Other potential biological roles can be seen in mice, where CD2AP is implicated in blood brain barrier function [43], and in Drosophila where the CD2AP ortholog *Cindr* is implicated in synaptic plasticity and Tau linked neurodegeneration [44, 45]. CD2AP’s role in AD biology has been predominantly examined in neurons, while our cell type enrichments point to the primary involvement of endothelial, oligodendrocyte, pericytes, and astrocytes. Future studies exploring the function of CD2AP in non-neuronal cells may prove useful in developing a broader perspective of CD2AP function in AD pathogenesis [6, 39].

### EED, Chr11

EED was identified by Kunkle et al., but not by three other major AD GWAS from the last few years [6, 7, 38, 39]. One possible explanation is that the EED locus contains PICALM, a known AD risk factor, and could lead EED to be overlooked by other types of studies. We do not believe that PICALM explains the TWAS risk identified here for a number of reasons. Colocalization analysis yielded a high probability (H4 *p* = 0.69) that the GWAS signal and EED functional association arise from shared causal variants. While there is a non-zero probability (H2 *p* = 0.18), our signal arises only due to GWAS association, which may be a function of EED risk variants being in linkage with PICALM risk variants (Additional file 2: Table S6). PICALM is positively associated with AD as it is involved in clathrin-mediated endocytosis of APP and subsequent generation of amyloid [46, 47]; however, EED’s TWAS association *Z* value is − 5.85 (Table 1). This infers that overexpression of the loci’s regulated gene target is protective against AD, and this means the valence of the effect runs opposite to PICALM’s. Given the colocalization results, it is possible that different AD risk variants within this region affect both EED and PICALM transcription and are affected by partial linkage. Alternatively, EED is a component of the polycomb repressive complex 2 (PRC2) that functions as a histone methylase depositing the repressive mark H3K27 [46–48]. Targeting EED and the PRC2 complex plays a role in synaptic plasticity, as genetic ablation of EED impacts long-term potentiation, a surrogate measure to hippocampal memory function [49]. Additionally, EED promotes neurogenesis within the hippocampus, potentially making the brain more resistant to age-related neurodegenerative changes [50, 51]. Interestingly, the EED-expanded gene set was enriched for many processes involving translation and RNA splicing, two biological domains that would be impacted by heterochromatin regulation (Additional file 1: Fig. S.9). This association remains interesting given the potential roles of both EED and PICALM in AD biology, and further study is needed to fully understand the roles of each gene’s contribution to the disease risk.

### MTCH2, Chr11

MTCH2, or mitochondrial carrier 2, is a SLC25 family member of transporters, which localizes to the inner mitochondrial membrane. MTCH2 has been identified as an AD risk factor [6, 38]. Previous studies implicated risk variants regulating SPI1 [52], a well-known microglial transcription factor shown to interact with known AD risk genes including TREM2, and CELF1 expression, with fine mapping potentially implicating CELF1 [53] from the MTCH2 locus in AD. Knockdown of SPI1 in microglia have implicated its role in regulating of microglial genes involved in phagocytotic activity driving AD as a potential target for treatment. MTCH2 is negatively associated with AD (*Z* value = − 5.73 Table 1); the direction of this effect indicates that reduced expression of MTCH2 increases AD pathology. This reinforces that MTCH2 is the associated gene over SPI1, as knockdown of SPI1 reduced AD pathology. Colocalization analysis of this target feature showed a high probability (H4 *p* = 0.993) that MTCH2 functional association and GWAS signal arose from shared causal variants. This adds confidence GWAS signal from previous AD studies are acting through MTCH2. The biological role of MTCH2 in the brain is unclear. MTCH2 is known to contribute to adipocyte function and regulation of lipid metabolism [52, 54] and to be genetically associated with obesity [55]. However, MTCH2 clearly has a role outside of adipocytes, as inhibition of MTCH2 increases products of metabolism, such as pyruvate and pyruvate dehydrogenase [56] in both the brain and muscle. Our gene set enrichment analysis further supports MTCH2 involvement across a wide array of metabolic processes (Additional file 1: Fig. S.10).

MTCH2 knockout mice exhibited deficits in both metabolic processes and hippocampal dependent spatial learning tasks [54, 57, 58]. There are known links between nutrition, specifically cholesterol consumption levels, in AD [59], relevant to health risks of cardiovascular function, another well-known risk factor for AD. Concordantly, there are associations observed between obesity [60] and AD, suggesting that MTCH2 variants associated with AD and obesity may be acting, at least in part, through a common mechanism [61]. This does not account for the spatial learning deficits, suggesting that this is not the whole answer. Yet, consistent with a non-neuronal locus of effect, we did observe that the MTCH2-expanded gene set is enriched for microglial and oligodendrocyte cell type markers (Fig. 3d). MTCH2 knockout mice exhibit elevated levels of microglia and diminished synaptic density in the basal forebrain, both of which could be explained by perturbations in microglial and oligodendrocyte function [58]. The mechanisms through which MTCH2 exerts its influence upon AD pathogenesis are currently not fully elucidated; additional studies will be necessary to fully understand the relevant biology.

### KNOP1, Chr16

KNOP1 is a lysine rich nucleolar protein lacking direct publications or much knowledge of its biological function; downregulation is associated with AD risk (Table 1). The KNOP1 gene resides within the IQCK locus, and Kunkel et al. [6] found linkage between KNOP1 and AD; however, none of the other recent GWAS studies found KNOP1 associated with AD. IQCK was a novel genome-wide locus from the Kunkle et al. [6] genetic meta-analysis. What data exists for KNOP1 suggests that it associates condensed chromatin during mitosis, which is partially supported by AP2M1 a KNOP1 yeast two-hybrid binding partner identified by the Human Reference Interactome (HuRI) [6, 62], and binds with a large number of H2B associated proteins [62, 63]. This preliminary evidence aligns with the KNOP1 expanded gene set enrichment which shows a strong signal for mitotic cell phase transition and regulation of DNA-binding transcription factor activity (Additional file 1: Fig. S.11). Intriguingly, the entire locus, similar to variants in the MTCH2 locus, is implicated in obesity [64, 65]. Colocalization analysis yielded a high probability that GWAS and functional associations were driven by shared genetic risk factors (H4 *p* = 0.809) (Additional file 2: Table S6). We hope that the finding that KNOP1 is associated with the AD risk will inspire future studies into its specific function.

### TREM2, Chr6

Identification of TREM2 in this study is consistent with previous work and knowledge in AD, as TREM2 is one of the most widely studied genes in Alzheimer’s disease, with links to both amyloid and tau pathology. TREM2 is expressed almost exclusively in the microglia, and it is a sentinel gene linking neuroinflammation to AD [66]. TREM2 null mutant mice crossed onto APP-PS1 AD transgenics exhibit deficits in microglial recruitment to amyloid plaques and increased spread of pathological tau [67]. Coding variants in TREM2 are estimated to confer a 2–4-fold increase in AD risk, higher than any gene other than APOE [68, 69]. Interestingly, APOE binds to TREM2, leading to activation and recruitment of the microglial cells, which elicits both phagocytic and proinflammatory responses [66]. While microglial cells seem to be represented in a greater proportion in AD brains, scRNA-Seq studies have found microglial populations to be a small fraction of total cell types regardless of disease status [31].TREM2 also binds to amyloid directly, with nanomolar affinity, and activates microglial clearance of amyloid deposition [67, 70]. Consistent with TREM2 function, our expanded TREM2 gene set expression was enriched for immune myeloid cellular lineages and cell process enrichments of immune activation and leukocyte migration (Fig. 3c, d, Fig. S12). Interestingly, our TREM2 *Z* score is − 4.52 (Table 1), which appears to contradict previous work. However, TWAS analysis associates only the genetic component of TREM2 expression, inferring that TREM2 genetic risk could function through disruption and dysregulation of TREM2 endogenous function. Furthermore colocalization analysis of this target yields a high probability (H1 *p* = 0.924) that this signal is driven from cis-regulatory variants affecting Trem2 expression but not contributing to the GWAS signal. This is consistent with the rare disruptive TREM2 coding variants and recessive loss of function associations with AD versus common expression modulating variants with lower effect sizes.

### APOC1 - CEACAM19 - CLPTM1, Chr19

APOC1, CLPTM1, and CEACAM19 were identified within this study, and all three genes reside within 600 Kb, less than a 1 Mb distance threshold, of each other. Due to this proximity, there appears to be complicated co-regulation in this region with upregulation of APOC1 and CEACAM19 associated with AD but downregulation of CLPTM1 associated with AD. Interestingly, all three of these genes were also identified in recent GWAS studies [7, 38]. This locus is particularly infamous for harboring the APOE genes, the largest known LOAD risk allele. APOE was the most coexpressed gene to APOC1, and this co-expressed pair had the highest average coexpression of any gene within a *cis*-locus to an associated gene, strongly supporting the co-regulation of APOC1 and APOE. Colocalization analysis for APOC1 and CEACAM19 exhibit a high probability (H4 of *p* = 0.996 and *p* = 1, respectively) that the GWAS signal from this region and the functional gene association are driven from different causal variants. This is likely a result of LD within this region (Additional file 2: Table S6). Little is known about the specific role of APOC1 in AD; however, as it is also a lipid carrier transport protein that, like APOE, is known to recruit the innate immune system, it may also have a role in regulation of microglial activation. More specifically, there are isolated studies which demonstrate linkage between APOC1 and declining cognition and memory in specific ethnic groups [71, 72]. Consistent with the APOC1 role in lipid transport and immune activation, our expanded cell process enrichments specifically identified phagocytosis and activation of immune response (Fig. 3b), supporting a shared role between APOE and APOC1 as the biological contributor to enhanced AD risk and making the disentanglement of each gene’s unique AD risk contribution that much more difficult.

While APOC1, CEACAM19, and CLPTM1 are associated with AD, both in this study and previous studies [72–74], a causal association to AD remains unclear. However, there is a small literature pointing to a role for CLPTM1 in the regulation of GABA receptor trafficking from the ER to the plasma membrane, suggesting that CLPTM1 could regulate inhibitory neurotransmission [75, 76]. Unlike APOC1 and CEACAM19, colocalization yields high probabilities that this association and GWAS signal arise from shared causal variants (H4 *p* = 0.359) as well as a high probability that this signal arises only from GWAS signal (H2 *p* = 0.574). While this speaks to the high LD within this window and the dominating signal from APOE, there remains evidence for a distinct regulatory schema for CLPTM1 (Additional file 2: Table S6). The regulation of GABA currents could be a synaptic scaling factor, adjusting the responsiveness of synaptic firing. Ge et al. [75] found that increasing CLPTM1 levels decreased miniature inhibitory postsynaptic potentials, while reciprocally decreasing CLPTM1 levels elevated GABA currents in the post-synaptic neuron, strongly suggesting that CLPTM1 negatively regulates GABAergic signaling. A recent literature meta-analysis looking at neurotransmitter synaptic dysregulation in AD found decreased levels GABA in AD patients, supporting the potential dysregulation of GABAergic signaling in AD [77].

## Conclusions

We have presented here a TWAS analysis of Alzheimer’s using weights trained from RNA-Seq expression values derived from 6 distinct cortical regions to associate genetic risk to expression differences in 404 cases and 231 controls. This methodology has shown its power in resolving additional mechanistic insights in the impact of risk variants on transcripts responsible for AD pathology. We provide a resource of trained expression weights for 6818 genes which is broadly abstractable across the neocortex and when used in combination with summary GWAS statistics can perform powerful associations across a broad range of neocortical phenotypes.

## Supplementary Information

**Additional file 1.** This file contains the supplementary figures (S1-S14) and the Supplementary Methods Sections 1-4.

**Additional file 2.** This file contains the supplementary tables (S1-S6).

## Data Availability

Weights and analysis files are available via the AD Knowledge Portal (https://adknowledgeportal.synapse.org). The AD Knowledge Portal is a platform for accessing data, analyses, and tools generated by the Accelerating Medicines Partnership (AMP-AD) Target Discovery Program and other National Institute on Aging (NIA)-supported programs to enable open-science practices and accelerate translational learning. The data, analyses, and tools are shared early in the research cycle without a publication embargo on secondary use. Data is available for general research use according to the following requirements for data access and data attribution (https://adknowledgeportal.synapse.org/DataAccess/Instructions). For information about data and data sources, see (https://adknowledgeportal.synapse.org/Explore/Studies/DetailsPage?Study=syn22313785)

## Abbreviations

AD: Alzheimer’s disease
CEU: Utah residents (CEPH) with Northern and Western European ancestry
FDR: False discovery rate
LOAD: Late-onset Alzheimer’s disease
EOAD: Early-onset Alzheimer’s disease
GWAS: Genome-wide association studies
TWAS: Transcriptome-wide association study
DLPFC: Dorsolateral prefrontal cortex
TCX: Temporal cortex
PFC: Prefrontal cortex
STG: Superior temporal cortex
IFG: Inferior temporal gyrus
PHG: Parahippocampal gyrus
AMP-AD: Accelerating Medicines Partnership - Alzheimer’s Disease
CMC: CommonMind Consortium
JCP: Joint probability correlation
SMR: Summary Mendelian randomization
GPR: G-protein coupled receptor
ILV: Intraluminal vesicles
